# Complete Genome Sequence of a Tobacco-Infecting, Tomato-Blistering Mosaic Virus

**DOI:** 10.1128/genomeA.00701-14

**Published:** 2014-08-07

**Authors:** Fernando Lucas Melo, Jhon Eric Alves Fernandes, Bergmann Morais Ribeiro, Simone Graça Ribeiro

**Affiliations:** a Universidade de Brasília, Brasília, Brazil; b Embrapa Recursos Genéticos e Biotecnologia, Brasília, Brazil

## Abstract

The complete genome sequence of a new tomato-blistering mosaic virus (ToBMV) isolate was determined. This tymovirus isolate was first described infecting tobacco during the 1980s, but it also infects other *Solanaceae* members experimentally. The genome has 6,257 nucleotides and shares 88% nucleotide identity with the ToBMV isolated from tomato.

## GENOME ANNOUNCEMENT

The tymoviruses (family *Tymoviridae*, genus *Tymovirus*) are icosahedric, non-enveloped, single-stranded positive sense RNA viruses (1). To date, only five tymoviruses infecting different plant species were reported in Brazil: (i) cassia yellow mosaic-associated virus (2), (ii) eggplant mosaic virus (EMV) (3, 4), (iii) passion fruit yellow mosaic virus (5), (iv) petunia vein banding virus (6), and (v) tomato blistering mosaic virus (ToBMV) (7). Most of these viruses were identified as distinct tymoviruses based on biological, biochemical, and serological properties, and no complete genomic RNA sequences are available for these viruses, except for the recently described ToBMV (7) (GenBank accession no. KC840043). This tentative new tymovirus species (ToBMV), isolated from tomato, was serologically related to EMV, suggesting a cryptic diversity within Brazilian tymoviruses. Therefore, we revisited the previously described tobacco-infecting EMV (3) to confirm its taxonomic status.

Frozen viral stock was recovered by mechanical inoculation in *Nicotiana benthamiana* and infected plants were used to rub-inoculate *N. tabacum* ‘TNN’ plantlets. The virus was purified from symptomatic tobacco leaves as previously described (3). The viral RNA was extracted according to Chang et al. (8), submitted to agarose electrophoresis, and a band corresponding to a viral genome size of approximately 7 kb was extracted, and the RNA was purified using an Ultrafree-DA centrifugal filter device (Millipore). The viral RNA was sequenced at Macrogen (South Korea) using an Illumina HiSeq 2000 platform. The paired-end reads were assembled using CLC Genomics Workbench version 6.0.3. The assembled contigs were submitted to a BLASTx search (9) against a local viral genome database. A contig of 6,256 nucleotides was found to be similar to tomato-blistering mosaic virus. This contig was annotated and tree intact open reading frames (ORFs) were identified. The ORF1 (5427 nt) encodes the replication polyprotein with 1809 amino acids. The ORF2 (1956 nt) encodes the movement protein and the ORF3 (573 nt) encodes the capsid protein, with 652 and 191 amino acids, respectively. The 5′ and 3′ untranslated regions (UTRs) are 129 and 117 nt long. Moreover, a phylogenetic analysis using 24 complete genomes available in GenBank confirmed that the previously identified EMV-tobacco isolate is, in fact, an isolate of ToBMV, with 88% nucleotide identity over the entire genome. Interestingly, these two ToBMV isolates differ in the ability to infect *N. tabacum* ‘TNN’ plants (3, 7). The comparison of the coding region of both isolates revealed that most of the nucleotide differences among the two genomes are synonymous, except for those occurring in the ORF2, which presented a high degree of non-synonymous substitution, warranting further investigation of the role of this protein in infection and host adaptation.

### Nucleotide sequence accession number.

The nucleotide sequence has been deposited in GenBank under the accession no. KJ940970.
